# Plasma Homocysteine as a Potential Marker of Early Renal Function Decline in IgA Nephropathy

**DOI:** 10.3389/fmed.2022.812552

**Published:** 2022-03-07

**Authors:** Yan-Na Wang, Han Xia, Zhuo-Ran Song, Xu-Jie Zhou, Hong Zhang

**Affiliations:** ^1^Renal Division, Peking University First Hospital, Beijing, China; ^2^Peking University Institute of Nephrology, Beijing, China; ^3^Key Laboratory of Renal Disease, Ministry of Health of China, Beijing, China; ^4^Key Laboratory of Chronic Kidney Disease Prevention and Treatment (Peking University), Ministry of Education, Beijing, China; ^5^Renal Division, Xingtai City People's Hospital, Xingtai, China

**Keywords:** IgA nephropathy, homocysteine, chronic kidney disease, primary glomerular diseases, kidney disease progression

## Abstract

Hyperhomocysteinemia (HHcy) is very common among patients with chronic kidney disease (CKD), and related to the risk of cardiovascular events and mortality in these patients. However, the prevalence of HHcy in primary causes of CKD and its role in kidney disease progression are not well-understood. In this study, we investigated the prevalence of HHcy in different CKD stages in 221 patients with IgA nephropathy (IgAN) and 194 patients with other primary glomerular diseases. We also evaluated the association of homocysteine (Hcy) [after adjusted for estimated glomerular filtration rate (eGFR)] with CKD progression event, defined as ESKD or 50% decline in eGFR, in a cohort of 365 patients with IgAN. The prevalence of HHcy was 67.9% (150/221), 53.5% (76/142), 51.5% (17/33), and 42.1% (8/19) in patients with IgAN, membranous nephropathy, minimal change disease, focal segmental glomerulosclerosis, respectively. The Hcy/eGFR ratio was significantly associated with pathologic features of IgAN, including the proportion of global glomerulosclerosis (*r* = 0.38, *p* < 0.001), the proportion of ischemia originated glomerular sclerosis (*r* = 0.32, *p* < 0.001), and the severity of tubular atrophy/interstitial fibrosis (*r* = 0.57, *p* < 0.001). Importantly, Hcy/eGFR ratio was an independent risk factor for CKD progression event (hazard ratio, 1.38; 95% confidence interval, 1.13–1.68; *p* = 0.002). The risk of CKD progression events continuously increased with the Hcy/eGFR ratio, but reached a plateau when Hcy/eGFR ratio was >1.79. Our findings suggest that elevated Hcy/eGFR ratio may be an early marker of poor renal outcome in IgAN.

## Introduction

Primary glomerular diseases (PGD)—glomerular diseases that are not caused by a systemic disease—account for about 20% of chronic kidney disease (CKD) in most countries (1). A great number of PGD are “silent” at onset of the disease, and are diagnosed on urinary tests during routine medical examination (2). Unlike other causes of CKD such as diabetes and hypertension, they frequently affect young people with associated morbidity and high cost. IgA nephropathy (IgAN) is the most common PGD worldwide and the most common cause of ESKD among Asian populations (3). IgAN is currently monitored by the presence of proteinuria and/or changes in serum creatinine indicating decline in glomerular filtration rate (GFR). However, the lack of reproducibility in albuminuria measurements and high variability in GFR at mild to moderate stages of disease have raised concerns about their ability to accurately represent CKD progression.

Homocysteine (Hcy) is a sulfur-containing intermediary amino acid produced following the metabolic conversion of methionine to cysteine. Hyperhomocysteinemia (HHcy) is a condition characterized by the increase in plasma levels of Hcy—total plasma levels of Hcy >15 μmol/L. Hcy can reduce the utilization activity of nitric oxide, increase oxidative stress, induce endothelial dysfunction, and promote the proliferation of vascular smooth cells (4, 5). Raised plasma total Hcy is an independent risk factor for cardiovascular disease and atherothrombosis (6). Patients with CKD have markedly raised Hcy, and the risk of cardiovascular events and mortality is related to the concentration of Hcy (7). Folic acid therapy could significantly delay the progression from normal to moderate CKD in patients with hypertension (8). However, the role of Hcy levels in disease progression in patients with PGD remains unclear.

In this study, we enrolled 221 cases with IgAN and 194 cases with other PGD to investigate the prevalence of HHcy in different CKD stages in these patients. We also evaluated the association of Hcy/eGFR ratio with disease severity and CKD progression in a cohort of 365 patients with IgAN.

## Materials and Methods

### Study Participants

The cross-sectional study was based on data obtained from medical records from the renal division of Peking University First Hospital. We included participants with PGD who underwent comprehensive health examinations, including renal biopsy and plasma concentrations of total Hcy from January 2016 to December 2020. We excluded individuals with gout, pyuria, or nephritis secondary to systemic disease or cancer, as well as other factors that influence the plasma Hcy levels. Individuals with gastrointestinal diseases, alcoholic addiction, vegetarian lifestyle, and regular consumption of folic acid and vitamin supplements were also excluded. Finally, 221 cases with IgAN were recruited in the observational study. The diagnosis was based on renal biopsy by light microscopy, immunofluorescence, and electron microscopy. At the same time interval, 142 patients with membranous nephropathy (MN), 33 patients with minimal change disease (MCD), and 19 patients with focal segmental glomerulosclerosis (FSGS) were enrolled served as disease controls.

In addition, to explore the role of Hcy in disease progression, a total of 365 patients with biopsy-proven IgAN diagnosed between 2009 and 2020 were selected from the prospective IgAN database at Peking University First Hospital. Patients in the database were followed up regularly every 3–6 months.

This study was approved by the Ethics Committee of Peking University First Hospital and informed written consent was obtained from all patients.

### Data Collection

Clinical manifestations at the time of renal biopsy, including age, sex, homocysteine, serum creatinine, 24-h urine protein excretion, uric acid, and other biochemical characteristics were collected from the medical records. The definition of HHcy was referred to as the presence of homocysteine concentrations >15 μmol/L. The eGFR was calculated using the Chronic Kidney Disease Epidemiology Collaboration (CKD-EPI) equation (9). The Oxford classification of IgAN was used for the evaluation of pathologic lesions for those with more than eight glomeruli in biopsy specimens (10). The CKD progression event was defined by a 50% decline in eGFR or ESKD that was confirmed by a second evaluation obtained at least 4 weeks later.

### Statistical Analyses

Continuous variables were presented as mean ± standard deviation for normally distributed variables or median with interquartile range for non-normally distributed variables. Categorical variables were summarized as frequency with percentage. For continuous variables, the significance of differences between groups was determined using independent sample *t*-test. A Mann-Whitney *U*-test was used to compare the differences between two independent samples when the sample distributions are not normally distributed. Differences among categorical variables were analyzed using the chi-squared test.

Cox proportional hazards models were adopted to evaluate the relationship between Hcy/eGFR ratio and risk of end point. Sex, age, eGFR, proteinuria, mean arterial pressure, and use of steroids and/or other immunosuppressive agents were adjusted in multivariable-adjusted Cox proportional hazards models. Receivers operating characteristic (ROC) curves were constructed at the most discriminating cut-off point value to predict the disease progression. Kaplan-Meier analysis was used to derive cumulative kidney survival curves. The division between the groups of participants was on the basis of the cut-off value in the ROC curves, and difference between curves was analyzed using a log-rank test. The association between Hcy/eGFR ratio and end point was also evaluated on a continuous scale with restricted cubic spline curves based on Cox proportional hazards models. Knots for the cubic splines were placed with default parameters.

To investigate correlations between Hcy/eGFR ratio and clinicopathological parameters in IgAN patients, Pearson's correlation coefficients were used for continuous variables and Spearman's correlation coefficients for binary and ordinal variables. Partial correlation analysis was used to quantify the correlation between two variables after removing the effects of other variables. All *p*-values were 2 tailed, and values < 0.05 were considered statistically significant. Analyses were performed using SPSS software v20.

## Results

### Association of Hyperhomocysteinemia With Reduced Renal Function

The characteristics of the patients with PGD at the time of kidney biopsy are described in Table 1. In the cross-sectional study, we randomly enrolled 221 cases with biopsy proven IgAN. The mean age of IgAN was 37.7 ± 11.6 years, and men accounted for 52.9% of the cohort. At the time of diagnosis, the average eGFR was 66.6 ± 32.5 mL/min per 1.73 m^2^ and the median proteinuria level was 1.4 g/24 h (interquartile range, 0.6–3.0 g/24 h).

**Table 1:** Baseline characteristics of patients with different primary glomerular diseases.

**Characteristics**	**IgAN** **(***n*** = 221)**	**MN** **(***n*** = 142)**	**MCD** **(***n*** = 33)**	**FSGS** **(***n*** = 19)**
Age (years)	37.7 ± 11.6	52.1 ± 16.1	46.0 ± 19.1	38.5 ± 15.4
Sex (men, %)	117 (52.9)	92 (64.8)	18 (54.5)	15 (78.9)
Homocysteine (μmol/L)	19.1 (14.1–27.2)	16.0 (10.2–24.8)	17.1 (9.6–27.1)	13.7 (11.6–27.4)
Serum creatinine (μmol/L)	111.0 (81.0–166.2)	91.8 (65.3–128.3)	90.9 (64.9–164.1)	127.5 (82.9–212.6)
eGFR (mL/min per 1.73 m^2^)	68.1 (40.2–91.0)	78.0 (47.2–100.8)	76.1 (36.8–109.6)	61.0 (29.0–88.1)
Proteinuria (g/24 h)	1.4 (0.6–3.0)	3.3 (1.3–7.0)	3.0 (1.0–7.1)	1.8 (0.5–7.0)
Serum uric acid (μmol/L)	403.7 ± 106.6	390.4 ± 116.0	411.9 ± 123.7	412.1 ± 96.9
Blood urea nitrogen (mmol/L)	8.2 ± 5.1	8.5 ± 5.2	8.5 ± 6.5	11.4 ± 9.2
Folic acid (nmol/L)	29.6 (18.1–53.9)	17.4 (13.3–52.2)	21.4 (12.8–53.5)	33.1 (17.5–53.5)
Vitamin B12 (pg/mL)	245.5 (166.3–337.0)	255.0 (186.0–355.0)	220.0 (174.5–442.8)	292.0 (148.5–639.5)
Treated with steroids or other immunosuppressives (*n*, %)	46 (20.8)	78 (54.9)	32 (97.0)	19 (100.0)

*Data are presented as n (%), mean ± SD, or median (25th−75th percentile). IgAN, IgA nephropathy; MN, membranous nephropathy; MCD, minimal change disease; FSGS, focal segmental glomerulosclerosis; eGFR, estimated glomerular filtration rate*.

The mean homocysteine level in IgAN was 23.2 ± 15.3 μmol/L (range, 7.1–100.0 μmol/L). HHcy was detected in 150 (67.9%) of the 221 patients in IgAN group. Whereas, the prevalence of HHcy was 53.5% (76/142), 51.5% (17/33), and 42.1% (8/19) in MN, MCD and FSGS groups, respectively. As shown in Supplementary Table 1, individuals with HHcy presented with more severe disease, including higher serum creatinine (*p* < 0.001) and uric acid levels (*p* < 0.001), and lower eGFRs (*p* < 0.001) than those without HHcy, which was not specific to any kind of PGD.

**Figure 1:**
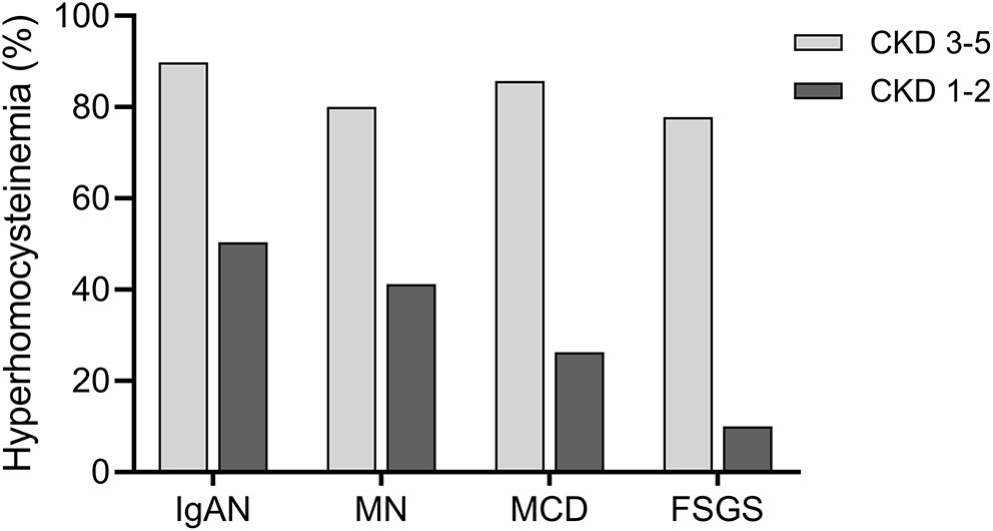
Prevalence of hyperhomocysteinemia (HHcy) in different CKD stages in primary glomerular diseases. In patients with CKD stages 3–5, the prevalence of HHcy was 89.8% (88/98), 80.0% (36/45), 85.7% (12/14), and 77.8% (7/9) in IgAN, MN, MCD, and FSGS, respectively. In patients with CKD stages 1–2, the prevalence of HHcy was 50.4% (62/123), 41.2% (40/97), 26.3% (5/19), and 10.0% (1/10) in IgAN, MN, MCD, and FSGS, respectively.

We also explored the prevalence of HHcy in different CKD stages (CKD stages 1–2 vs. 3–5) in IgAN and disease controls (Figure 1). In CKD stages 1–2, the prevalence of HHcy was 50.4, 41.2, 26.3, and 10.0% in IgAN, MN, MCD, and FSGS, respectively (*p* = 0.02). In CKD stages 3–5, the prevalence of HHcy was 89.8, 80.0, 85.7, and 77.8% in IgAN, MN, MCD, and FSGS, respectively (*p* = 0.38). Specifically, the prevalence of HHcy in CKD stages 1–2 in IgAN was 36.2% (21/58) and 63.1% (41/65), respectively. Thus, HHcy is very common in IgAN cases of normal or mildly reduced renal function (about 1/3 in CKD 1 and 2/3 in CKD 2), which may be of clinical importance. In line with expectations, the prevalence increased with decreasing renal function, which was 84.4% (54/64), 100.0% (28/28), and 100.0% (6/6) in CKD stages 3, 4, and 5 in IgAN, respectively (Supplementary Table 2).

### Association of Hyperhomocysteinemia With Clinicopathologic Characteristics in IgAN

**Figure 2:**
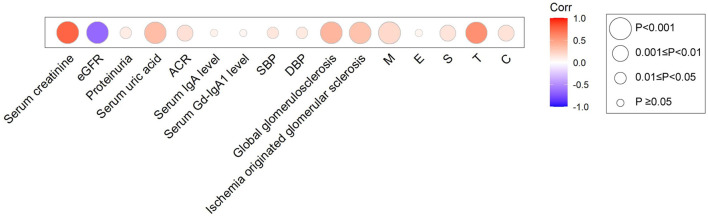
Heatmap for the association between Hcy/eGFR ratio and clinical and pathologic manifestations in IgAN. Circles were colored by the correlation coefficients, and the size of the circle represents correlation significance level.

Then, we explored the correlations between Hcy (after adjusted for eGFR) and clinicopathological parameters in patients with IgAN (Figure 2, Supplementary Table 3). The Hcy/eGFR ratio showed a negative correlation with eGFR (*r* = −0.65, *p* < 0.001), and remained significant after controlling for cardiovascular risk factors, including age, sex, hypertension, and total cholesterol levels (*r* = −0.63, *p* < 0.001). The Hcy/eGFR ratio was also positively correlated with serum creatinine (*r* = 0.76, *p* < 0.001), serum uric acid (*r* = 0.35, *p* < 0.001), proteinuria (*r* = 0.10, *p* = 0.03), systolic blood pressure (*r* = 0.13, *p* = 0.01), and diastolic blood pressure (*r* = 0.11, *p* = 0.03). In addition, the Hcy/eGFR ratio was significantly associated with pathologic features of IgAN, including the mesangial hypercellularity (*r* = 0.20, *p* < 0.001), the segmental glomerulosclerosis (*r* = 0.14, *p* = 0.01), the proportion of global glomerulosclerosis (*r* = 0.38, *p* < 0.001), the proportion of ischemia originated glomerular sclerosis (*r* = 0.32, *p* < 0.001), the severity of tubular atrophy/interstitial fibrosis (*r* = 0.57, *p* < 0.001), and the presence of crescent (*r* = 0.15, *p* = 0.01). These results suggest that Hcy might contribute to the progression of IgAN.

### Association of Hcy/EGFR Ratio With Kidney Disease Progression in IgAN

The clinical and pathologic characteristics of IgAN cohort are summarized in Supplementary Table 4. At baseline there were 199 (54.5%) men and mean age was 39.1 ± 12.7 years. eGFR was 70.6 ± 33.0 mL/min per 1.73 m^2^ and the median proteinuria level was 1.1 g/24 h (interquartile range, 0.6–2.4 g/24 h). The mean homocysteine level in IgAN was 23.7 ± 19.4 μmol/L (range, 4.6–140.9 μmol/L). Overall, 238 participants (65.2%) received steroids or other immunosuppressive agents. After a median follow-up of 34 months (IQR, 22–49 months), 32 (8.8%) participants reached the composite kidney disease progression event, including 27 kidney failure events. Compared with other patients, these patients showed a higher prevalence of HHcy (90.6 vs. 64.3%; *p* = 0.003) and more severe histological lesions, including higher prevalence of M1 (65.6 vs. 43.2%; *p* = 0.02), S1 (87.5 vs. 58.9%; *p* = 0.001), and T1/T2 (96.9 vs. 38.7%; *p* < 0.001) according to the Oxford classification (Supplementary Table 5).

**Figure 3:**
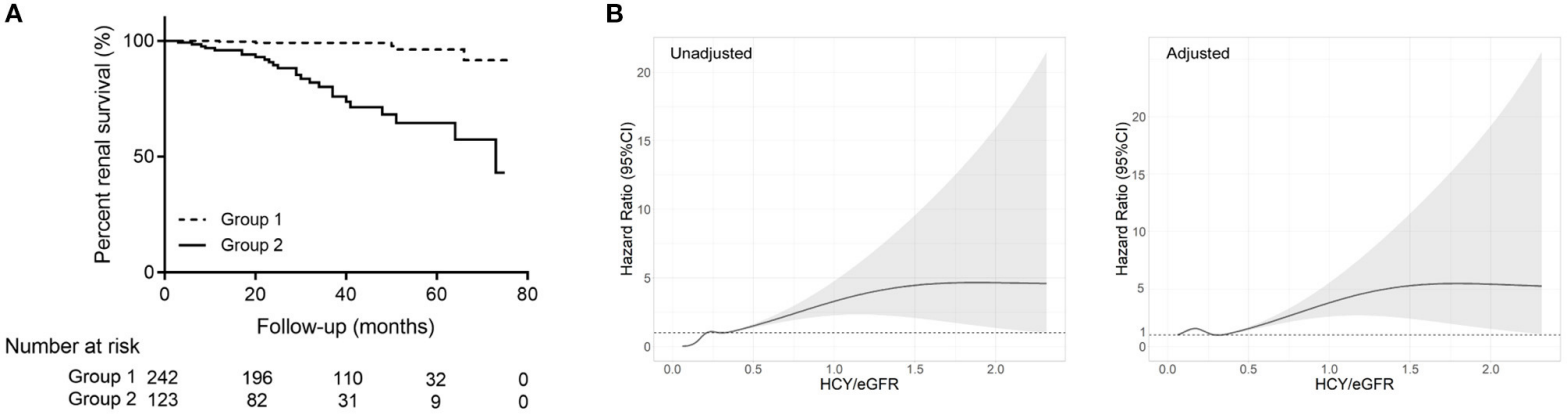
Association of Hcy/eGFR ratio with kidney disease progression in IgAN. **(A)** Kaplan-Meier kidney survival curves of participants with IgAN according to Hcy/eGFR ratio. The time zero was kidney biopsy. The division between the groups of participants was on the basis of the cut-off value of Hcy/eGFR ratio (Group 1: low Hcy/eGFR ratio; Group 2: high Hcy/eGFR ratio). **(B)** Unadjusted and multivariable-adjusted hazard ratios for CKD progression events according to Hcy/eGFR ratio on a continuous scale. The solid lines represent the estimated hazard ratios, with the shaded areas showing 95% confidence intervals derived from restricted cubic spline regressions with five knots. Reference lines for no association are indicated by the dashed lines at a hazard ratio of 1.0. The model was adjusted for age, sex, eGFR, proteinuria, mean arterial pressure, and use of steroids and/or other immunosuppressive agents.

As shown in Table 2, in cause-specific hazards models, Hcy/eGFR ratio was an independent risk factor for the composite kidney disease progression event in model 3 [hazard ratio (HR), 1.38; 95% confidence interval (95% CI), 1.13–1.68; *p* = 0.002] after adjustment for sex, age, baseline eGFR, proteinuria, mean arterial pressure, and use of steroids or other immunosuppressive agents. The ROC curves showed discriminatory power of Hcy/eGFR ratio on disease progression (Supplementary Figure 1). The area under the ROC curves (AUC) was 0.79 with the cut-off point of 0.46. Then, Kaplan-Meier curves demonstrated that patients with Hcy/eGFR ratio >0.46 had a higher incidence of the composite kidney disease progression event (log-rank test, *p* < 0.001) (Figure 3A).

**Table 2:** Hcy/eGFR ratio as a risk factor for the composite kidney disease progression event in IgAN.

	**Hazard ratio for composite outcome (95% confidence interval);** ***p***			
	**Unadjusted**	**Model 1^b^**	**Model 2^c^**	**Model 3^d^**
CKD progression event^a^, per 1 s.d. Hcy/eGFR	1.41 (1.27–1.58) 8.11 × 10^−10^	1.46 (1.30–1.65) 5.14 × 10^−10^	1.23 (1.06–1.42) 0.007	1.38 (1.13–1.68) 0.002

a *CKD progression event was defined as a 50% decline in eGFR or ESKD*.

b *Model 1 was adjusted for sex and age. Sex was analyzed as dichotomous data*.

c *Model 2 was adjusted for covariates in model 1 plus eGFR, proteinuria, and mean arterial pressure*.

d *Model 3 was adjusted for covariates in model 2 plus steroids or other immunosuppressive agents. Use of treatment with steroids and/or other immunosuppressive agents was analyzed as dichotomous data*.

The association between Hcy/eGFR ratio and end point was also evaluated on a continuous scale with restricted cubic spline curves based on Cox proportional hazards models (Figure 3B). The risk of CKD progression events continuously increased with the Hcy/eGFR ratio, but reached a plateau when Hcy/eGFR ratio was >1.87. The non-linear association was also found in the multivariable-adjusted model, with a plateau when Hcy/eGFR ratio was >1.79.

## Discussion

In this study, we investigated the prevalence of HHcy in 221 patients with IgAN and 194 patients with other primary glomerular diseases, and found that patients with HHcy tended to present with more severe disease than those without HHcy. Among the four kinds of PGD, IgAN had the highest prevalence of HHcy, especially in patients with CKD stages 1–2. Notably, our study demonstrated that Hcy/eGFR ratio was associated with the chronic changes in kidney biopsy specimens in IgAN. On the basis of our follow-up data, we found that elevated Hcy/eGFR ratio was independently associated with increased risk of kidney disease progression in IgAN.

The prevalence of HHcy in patients with CKD has been reported (11). Nevertheless, the different primary causes of CKD have not been considered in these analyses. The present study found that HHcy was most pronounced in patients with IgAN, of which 67.9% with Hcy > 15 μmol/L. Furthermore, the higher Hcy levels in IgAN were not due to gender distributional differences: men accounted for 52.9% of IgAN, the lowest among the four primary glomerular diseases. The association between Hcy and IgAN was independent of eGFR, as there was no significant difference in eGFR between IgAN and disease controls. Consistent with previous data, we found that patients with advanced CKD had an increased prevalence of HHcy. In addition, the prevalence differences of HHcy among primary glomerular diseases mainly existed in the early stages of CKD.

Moreover, we found that Hcy/eGFR ratio was significantly associated with eGFR, proteinuria, and multiple chronic pathologic features of IgAN, which are important markers for the development and progression of CKD. Thus, the Hcy/eGFR ratio may be a potential marker for predicting the prognosis of IgAN. Consistently, participants reached the CKD progression event presented more severe histological lesions, including mesangial hypercellularity, segmental glomerulosclerosis, and tubular atrophy/interstitial fibrosis. Chronic changes in kidney biopsy specimens have a major bearing on predicting prognosis of CKD and guiding treatment (12). Endocapillary hypercellularity is one of the pathologic features of IgAN that was found to be associated with disease severity (13). Repeat biopsies indicated that the progression of glomerular sclerosis is dependent on the degree of glomerular endothelial proliferation at the first biopsy (14). Chronic elevated plasma Hcy level alters functions of the vascular endothelial cells, which compromises the integrity of the vessel wall and in turn the vascular tone, leading to vascular inflammation (15). Therefore, early intervention of HHcy in IgAN is essential to prevent progression to CKD and ESKD, especially for those with glomerular endothelial injury.

A previous study has reported the association between Hcy levels and renal function decline in hypertensive adults (16). To our knowledge, we first estimated the role of Hcy in kidney disease progression in patients with IgAN. The Hcy/eGFR ratio was independently associated with the composite kidney disease progression event in IgAN during the follow-up. Similar results were obtained based on restricted cubic spline curves, which allowed for flexibility examining in the association between continuous Hcy/eGFR ratio and the risk of CKD progression events. However, our study has several limitations. First, this was a single-center study with relatively small sample size, and the findings need to be confirmed in other populations. Second, the follow-up was relatively short (with a median of ~3 years), during which few end points were observed.

In conclusion, HHcy was more prevalent in IgAN patients than in patients with other primary glomerular diseases, especially in the early stages of CKD. Hcy/eGFR ratio was significantly associated with markers of poor renal outcome in IgAN. Importantly, we use a cohort study demonstrating that elevated Hcy/eGFR ratio was independently associated with increased risk of disease progression in IgAN. Further high-quality trials of early intervention of HHcy in IgAN to prevent progression to CKD and ESKD are warranted.

## Data Availability Statement

The raw data supporting the conclusions of this article will be made available by the authors, without undue reservation.

## Ethics Statement

The studies involving human participants were reviewed and approved by the Ethics Committee of Peking University First Hospital. The patients/participants provided their written informed consent to participate in this study.

## Author Contributions

X-JZ and HZ designed the study. Y-NW carried out experiments and data analysis. HX and Z-RS contributed to sample collection and performed clinical characterization. All authors contributed to the manuscript and approved the submitted version.

## Funding

This work was supported by the National Natural Science Foundation of China (81970613, 82022010, 82131430172, and 82070733), Natural Science Foundation of Beijing Municipality (Z190023), Chinese Academy of Medical Sciences Research Unit (2019RU023), Clinical Medicine Plus X-Young Scholars Project of Peking University (PKU2020LCXQ003), Fok Ying Tung Education Foundation (171030), and Beijing Nova Program Interdisciplinary Cooperation Project (Z191100001119004).

## Conflict of Interest

The authors declare that the research was conducted in the absence of any commercial or financial relationships that could be construed as a potential conflict of interest.

## Publisher's Note

All claims expressed in this article are solely those of the authors and do not necessarily represent those of their affiliated organizations, or those of the publisher, the editors and the reviewers. Any product that may be evaluated in this article, or claim that may be made by its manufacturer, is not guaranteed or endorsed by the publisher.
