# Supplementary material for: Plasma Homocysteine as a Potential Marker of Early Renal Function Decline in IgA Nephropathy
Source: Front Med (Lausanne). 2022 Mar 7;9:812552. doi: 10.3389/fmed.2022.812552 (PMC8936167; doi:10.3389/fmed.2022.812552)

Supplementary Material

**Supplementary Table 1.** Comparison of demographic and clinical characteristics in patients with or without HHcy

| Characteristics | Hcy≤15 μmol/L  (*n* = 164) | Hcy >15 μmol/L  (*n* = 251) | *p* |
| --- | --- | --- | --- |
| Age (years) | 43.8±15.5 | 43.0±15.6 | 0.60 |
| Sex (men, %) | 73 (44.5) | 169 (67.3) | <0.001 |
| Homocysteine (μmol/L) | 10.9±2.6 | 29.7±17.4 | <0.001 |
| Serum creatinine (μmol/L) | 85.8±40.1 | 170.5±124.8 | <0.001 |
| eGFR (mL/min per 1.73m^2^) | 88.0±26.0 | 56.3±32.0 | <0.001 |
| Proteinuria (g/24h) | 2.1 (0.7-4.9) | 1.7 (0.8-4.2) | 0.14 |
| Serum uric acid (μmol/L) | 352.6±90.0 | 431.4±112.2 | <0.001 |
| Blood urea nitrogen (mmol/L) | 6.0±2.6 | 10.1±6.3 | <0.001 |
| Urinary albumin/creatinine ratio (mg/g) | 1276.0 (429.8-3334.2) | 827.0 (380.5-2340.6) | 0.15 |
| Urinary NAG (U/L) | 47.0 (27.0-69.3) | 39.0 (21.0-55.0) | 0.16 |
| Urinary A1M (mg/L) | 23.3 (8.9-40.1) | 25.9 (14.1-53.9) | 0.53 |
| C-reactive protein (mg/L) | 1.0 (0.3-2.4) | 1.2 (0.4-3.2) | 0.42 |
| Hemoglobin (g/L) | 128.3±20.5 | 121.9±24.8 | 0.03 |
| Platelet count (×10^9^/L) | 231.5±66.1 | 217.8±61.7 | 0.02 |
| Folic acid (nmol/L) | 31.8±19.2 | 28.7±17.6 | 0.40 |
| Vitamin B12 (pg/mL) | 427.7±320.2 | 236.0±104.7 | <0.01 |

Data are presented as *n* (%), mean ± SD, or median (25th-75th percentile). eGFR, estimated glomerular filtration rate; NAG, N-acetyl-β-D glucosaminidase; A1M, α1-microglobulin.

**Supplementary Table 2.**  The plasma levels of Hcy and the Hcy/eGFR ratio in different CKD stages

|  | CKD 1 (n=58) | CKD 2 (n=65) | CKD 3 (n=64) | CKD 4 (n=28) | CKD 5 (n=6) |
| --- | --- | --- | --- | --- | --- |
| Homocysteine (μmol/L) | 15.5±8.8 | 22.8±17.3 | 26.2±14.8 | 30.2±12.8 | 37.8±25.2 |
| HHcy (%) | 21 (36.2) | 41 (63.1) | 54 (84.4) | 28 (100.0) | 6 (100.0) |
| Hcy/eGFR, median, IQR | 0.13 (0.09-0.19) | 0.22 (0.18-0.33) | 0.49 (0.38-0.73) | 1.23 (1.02-1.63) | 2.88 (2.36-5.09) |

**Supplementary Table 3.** Association between Hcy/eGFR ratio and clinical and pathologic manifestations in IgA nephropathy

| Characteristics | r | *p* |
| --- | --- | --- |
| Serum creatinine | 0.76 | < 0.001 |
| eGFR | -0.65 | < 0.001 |
| Proteinuria | 0.10 | 0.03 |
| Serum uric acid | 0.35 | < 0.001 |
| Urinary albumin/creatinine ratio | 0.16 | 0.01 |
| Serum IgA level | 0.07 | 0.42 |
| Serum Gd-IgA1 level | 0.05 | 0.58 |
| SBP | 0.13 | 0.01 |
| DBP | 0.11 | 0.03 |
| The proportion of global glomerulosclerosis | 0.38 | < 0.001 |
| The proportion of ischemia originated glomerular sclerosis | 0.32 | < 0.001 |
| Oxford classification ^a^ |  |  |
| Mesangial hypercellularity | 0.20 | < 0.001 |
| Endocapillary proliferation | 0.06 | 0.29 |
| Segmental glomerulosclerosis | 0.14 | 0.01 |
| Tubular atrophy/interstitial fibrosis | 0.57 | < 0.001 |
| Crescent | 0.15 | 0.01 |

eGFR, estimated glomerular filtration rate; SBP, systolic blood pressure; DBP, diastolic blood pressure.

^a^ Oxford classification was developed by the Working Group of the International IgA Nephropathy Network and the Renal Pathology Society.

Pearson’s correlation coefficients were used to explore relationships between continuous variables. Spearman's correlation coefficients were applied for binary and ordinal variables.

**Supplementary Table 4.** Characteristics of participants in the Peking University First Hospital IgA nephropathy cohort

| Characteristics | Value (n = 365) |
| --- | --- |
| Age (years), mean±s.d. | 39.1±12.7 |
| Sex (men, %) | 199 (54.5) |
| SBP (mmHg), mean±s.d. | 127.1±15.2 |
| DBP (mmHg), mean±s.d. | 79.6±10.9 |
| Serum creatinine (μmol/l), mean±s.d. | 125.8±68.9 |
| eGFR (ml/min per 1.73m^2^), mean±s.d. | 70.6±33.0 |
| Proteinuria (g/24h), median, IQR | 1.08 (0.58 to 2.42) |
| Serum IgA level (g/L), mean±s.d. | 3.49±1.21 |
| Serum Gd-IgA1 (U/mL), mean±s.d. | 324.0±46.1 |
| Oxford classification ^a^ |  |
| M1 (%) | 165 (45.2) |
| E1 (%) | 125 (34.2) |
| S1 (%) | 224 (61.4) |
| T1-T2 (%) | 160 (43.8) |
| C1-C2 (%) | 236 (64.7) |

SBP,systolic blood pressure; DBP, diastolic blood pressure; eGFR, estimated glomerular filtration rate.

^a^ M1, mesangial hypercellularity score > 0.5; E1, the presence of endocapillary proliferation; S1, segmental glomerulosclerosis/adhesion; T, severity of tubular atrophy/interstitial fibrosis (T1: 26%-50%, T2 > 50%); C, presence of crescent (C1: < 25%, C2: 25%-100%). Oxford classification was developed by the Working Group of the International IgA Nephropathy Network and the Renal Pathology Society.

**Supplementary Table 5.** The follow-up characteristics of participants without or reached CKD progression event

|  | Reach the CKD progression event | |  |
| --- | --- | --- | --- |
| Characteristics | No (n = 333) | Yes (n = 32) | *p* |
| HHcy (%) | 214 (64.3) | 29 (90.6) | 0.003 |
| Hcy/eGFR, median, IQR | 0.26 (0.13 to 0.53) | 0.94 (0.46 to 1.66) | 0.02 |
| Oxford classification ^a^ |  |  |  |
| M1 (%) | 144 (43.2) | 21 (65.6) | 0.02 |
| E1 (%) | 118 (35.4) | 7 (21.9) | 0.12 |
| S1 (%) | 196 (58.9) | 28 (87.5) | 0.001 |
| T1-T2 (%) | 129 (38.7) | 31 (96.9) | < 0.001 |
| C1-C2 (%) | 218 (65.5) | 18 (56.3) | 0.11 |

eGFR, estimated glomerular filtration rate.

^a^ M1, mesangial hypercellularity score > 0.5; E1, the presence of endocapillary proliferation; S1, segmental glomerulosclerosis/adhesion; T, severity of tubular atrophy/interstitial fibrosis (T1: 26%-50%, T2 > 50%); C, presence of crescent (C1: < 25%, C2: 25%-100%). Oxford classification was developed by the Working Group of the International IgA Nephropathy Network and the Renal Pathology Society.

**Supplementary Figure 1.**  The ROC curves showed discriminatory power of Hcy/eGFR ratio on disease progression.


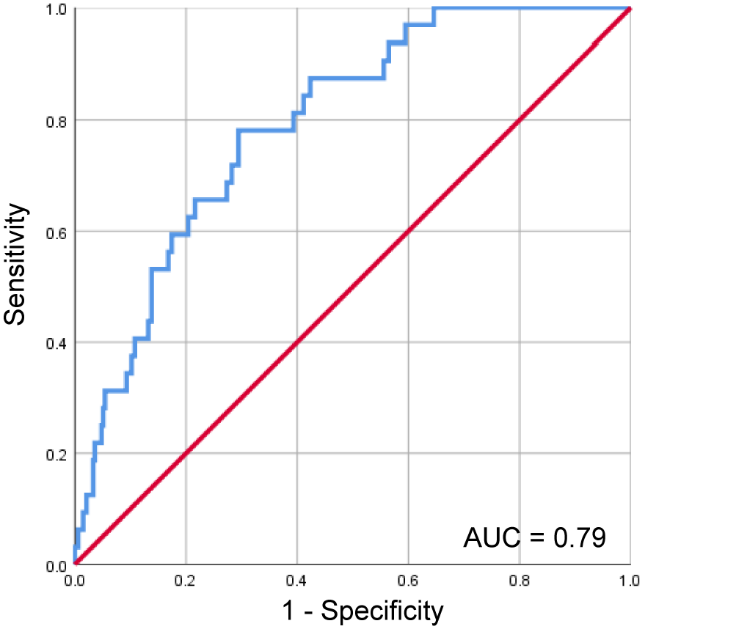

Supplement: Supplementary file 1 [file Data_Sheet_1.docx]
